# Supplementary material for: Distinct Mutational Profile of Lynch Syndrome Colorectal Cancers Diagnosed under Regular Colonoscopy Surveillance
Source: J Clin Med. 2021 Jun 1;10(11):2458. doi: 10.3390/jcm10112458 (PMC8198627; doi:10.3390/jcm10112458)
Supplement: Supplementary file 1 [file jcm-10-02458-s001.zip › jcm-1186885-supplementary.pdf]

SUPPLEMENTARY MATERIAL

FIGURES

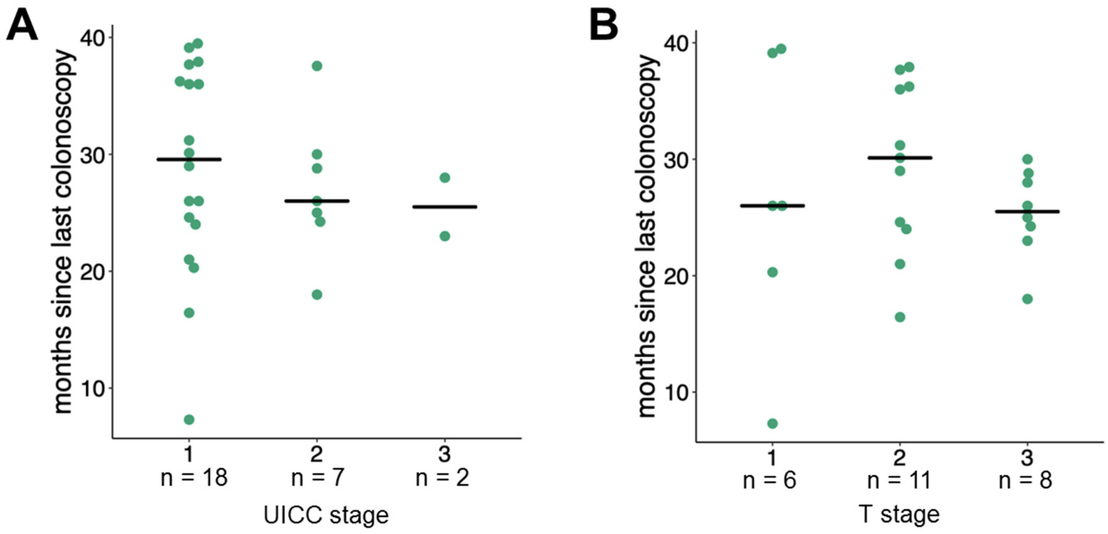

Supplementary Figure S1. Correlation analysis between time since last colonoscopy (in months) and the stage of detected tumor among incident CRCs. Neither UICC Stage (A), nor T stage (B) of detected tumors correlated with time since last colonoscopy (Wilcoxon Rank Sum Test, all p values not significant).

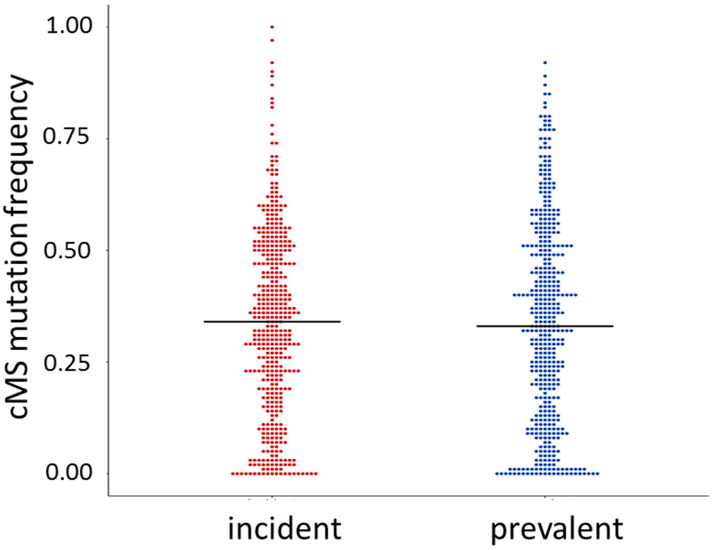

Supplementary Figure S2. CMS mutation frequencies in *MLH1*-associated incident and prevalent cancers.

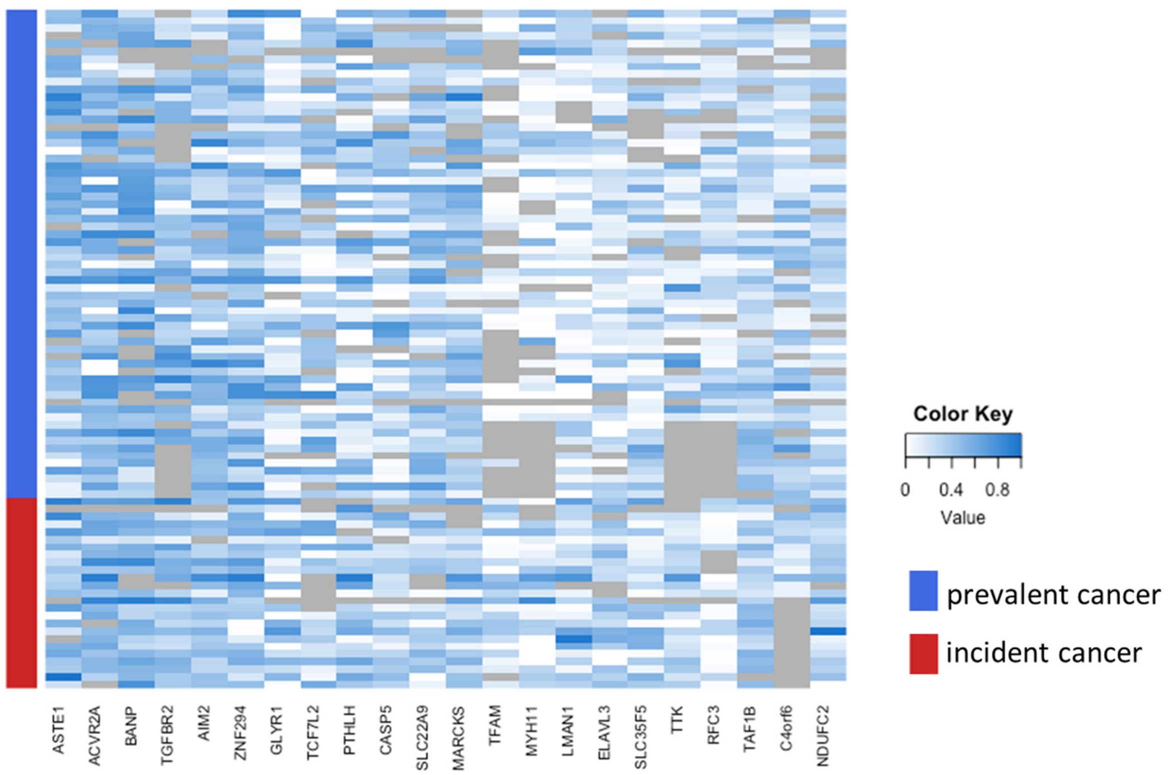

Supplementary Figure S3. Heat map of the relative frequency of mutant alleles is shown for 22 cMS (columns) in incident (rows marked red on the left side) and prevalent (rows marked blue on the left side) CRC obtained using ReFrame clustered in supervised manner. cMS mutation frequency is indicated by blue boxes of different intensity. Dark blue represents high mutation frequency, whereas pale blue represents low mutation frequency, and white absence of mutations (see color key). No specific clustering of cMS mutation frequencies could be observed in either of the CRC groups.

SUPPLEMENTARY MATERIAL

TABLES

Supplementary Table S1. Primer sequences.

Gene name	Forward primer	Reverse primer
ACVR2A	GTTGCCATTTGAGGAGGAAA	CAGCATGTTTCTGCCAATAATC
AIM2	TTCTCCATCCAGGTTATTAAGGC	TTAGACCAGTTGGCTTGAATTG
ASTE1	ATATGCCCCCGCTGAAATA	TTGGTGTGTGCAGTGGTTCT
BANP	TTCTGTGGAAGCTCTGCCTT	TCAAGTCGCATCAGATCCAG
C4orf6	CCAGAAGCAAATTCACAAGAC	TTTTGCGTGTTCCTTCCTTC
CASP5	CAGAGTTATGTCTTAGGTGAAGG	ACCATGAAGAACATCTTTGCCCAG
ELAVL3	GATGCGACCTGTTATCTCCAG	AGGTTGGTCTTGCTGTCGTC
GLYR1	GCCTCCAGAAGCTGTGACTT	ATCACCAACATCCCGTCATT
LMAN1	CACCCATGTCAGCTTTGCTA	GGAGGAATTTGAGCACTTTCA
MARCKS	GACTTCTTCGCCCAAGGC	GCCGCTCAGCTTGAAAGA
MYH11	CGGGGATTCTCTCTCTGTTTC	CTGAAGGCATGATACCTGGTG
NDUFC2	TGAATTTCAGGTTTGCATCG	AACATTTCACGGTCCCTCAC
PTHLH	TTTCACTTTCAGTACAGCACTTCTG	GAAGTAACAGGGGACTCTTAAATAATG
RFC3	TTTCTTTGTCCACAGACTCCATC	GTTACTTGCAATGGTGCTAATTTC
SLC22A9	GCGCCTACAGTGCCTACTCT	GCATGTGGAGCATTTCACAC
SLC35F5	TGTGGGGAAACTTACTGCAA	TCAAGTTTCAAACATCATATGCAA
TAF1B	ACCCAAATAAAAGCCCTCAAC	CTACTTAAAATTCCATTCCATGTCC
TCF7L2	GCCTCTATTCACAGATAACTC	GTTCACCTTGTATGTAGCGAA
TFAM	CTTTGGAAAAAGAAATCATGGAC	AACTATCCCACTTCTGCCTAACTG
TGFBR2	GCTGCTTCTCCAAAGTGCAT	CAGATCTCAGGTCCCACACC
TTK	TTCTTCATCCTCCAAGACTTTT	GATTTCCACAGGGATTCAAGA
ZNF294	AAGCCGAAGAGCTCATTGAA	CAGTTGTTAATTCCCAGCCTTC
B2M Exon 1	GGCATTCCTGAAGCTGACA	AGAGCGGGAGAGGAAGGAC
B2M Exon 2a	TTTCCCGATATTCCTCAGGTA	AATTCAGTGTAGTACAAGAG
B2M Exon 2b	TGTCTTTCAGCAAGGACTGG	CAAAGTCACATGGTTCACACG

Supplementary Table S2. Histopathological characteristics of incident cancers. The pattern of growth of the tumor was assessed in relation to the adjacent intact mucosa. A polypoid growth pattern is defined by a tumor having fibrovascular cores and a vertical growth that is more prominent than the transverse/horizontal growth whereas a flat growth pattern had a more prominent transverse/horizontal growth. Both polypoid and flat growth patterns were defined as tumors that were elevated above the level of the mucosa whereas a depressed growth pattern was defined as tumors that have the bulk of the tumor located below the level of the mucosa. HGD/CIS - high grade dysplasia/carcinoma in situ; n.a. not assessible.

Patient	Growth pattern	Grade and histology features	MMR deficient crypt foci
1	depressed	moderate	not identified
2	flat	moderate	not identified
3	flat	moderate with mucinous component	not identified
4	polypoid	moderate with mucinous component	not identified
5	flat	mucinous component	not identified
6	flat	moderate	present with adjacent HGD/CIS
7	n.a.	moderate with mucinous component	not identified
8	n.a.	moderate	not identified
9	n.a.	moderate	not identified
10	depressed	moderate with mucinous component	not identified
11	n.a.	moderate to poor with mucinous features and signet ring cells	not identified
12	flat	moderate with mucinous component	not identified
13	polypoid	moderate	not identified
14	flat	moderate to poor	not identified
15	flat	moderate	not identified
16	n.a.	moderate with mucinous component	not identified
17	flat	moderate	not identified
18	flat	moderate with mucinous component	not identified
19	polypoid	moderate with mucinous component	not identified
20	polypoid	moderate with mucinous component	not identified
21.a	flat	moderate with mucinous component	not identified
21.b	n.a.	moderate	not identified
22	depressed	moderate	present with adjacent HGD/CIS
23	depressed	moderate	not identified
24	flat	moderate with mucinous component	not identified
25	polypoid	moderate with mucinous component	not identified
26	polypoid	moderate with mucinous component	not identified
27	flat	moderate with mucinous component	not identified

Supplementary Table S3. Histopathological characteristics of prevalent cancers. The pattern of growth of the tumor was assessed in relation to the adjacent intact mucosa. A polypoid growth pattern is defined by a tumor having fibrovascular cores and a vertical growth that is more prominent than the transverse/horizontal growth whereas a flat growth pattern had a more prominent transverse/horizontal growth. Both polypoid and flat growth patterns were defined as tumors that were elevated above the level of the mucosa whereas an depressed growth pattern was defined as tumors that have the bulk of the tumor located below the level of the mucosa. n.a. not assessible.

Patient	Growth pattern	Grade and histology features	Patient	Growth pattern	Grade and histology features
1	n.a.	moderate with mucinous components	35	polypoid	moderate
2	depressed	moderate to poor	36	n.a.	moderate
3	polypoid	moderate	37	polypoid	well to moderate
4	polypoid	moderate with mucinous components	38	polypoid	moderate
5	n.a.	n.a.	39	n.a.	moderate to poor with mucinous components
6	n.a.	poor	40	n.a.	n.a.
7	polypoid	moderate	41	polypoid	moderate
8	flat	moderate with mucinous components	42	polypoid	moderate to poor with mucinous components
9	depressed	moderate	43	depressed	moderate with mucinous components
10	depressed	poor with mucinous components	44	depressed	poor
11	n.a.	moderate with mucinous components	45	depressed	n.a.
12	polypoid	moderate with mucinous components	46	n.a.	moderate to poor
13	n.a.	moderate to poor	47	n.a.	moderate
14	flat	moderate with mucinous components	48	depressed	moderate
15	depressed	moderate to poor	49	n.a.	moderate
16	polypoid	moderate to poor	50	depressed	moderate to poor with mucinous components
17	n.a.	moderate-poor	51	n.a.	moderate
18	depressed	n.a.	52	depressed	poor with mucinous components and signet ring cells
19	n.a.	n.a.	53	depressed	moderate
20	n.a.	n.a.	54	n.a.	poor
21	n.a.	n.a.	55	polypoid	poor with mucinous components
22	n.a.	moderate	56	n.a.	moderate to poor with mucinous components
23	n.a.	moderate	57	n.a.	moderate with mucinous components
24	n.a.	poor	58	polypoid	well to moderate
25	depressed	moderate	59	n.a.	moderate
26	depressed	poor with mucinous components	60	depressed	moderate to poor
27	n.a.	poor	61	polypoid	well to moderate
28	n.a.	poor	62	polypoid	moderate
29	flat	moderate	63	n.a.	moderate to poor
30	flat	moderate with mucinous components and signet ring cells	64	depressed	n.a.
31	flat	moderate with mucinous components	65	polypoid	well-moderate with mucinous components
32	depressed	moderate with mucinous	66	n.a.	n.a.

		components			
33	n.a.	n.a.	67	n.a.	well-moderate
34	n.a.	poor			

Supplementary Table S4. Summary of the mutation status in key CRC genes. Grey cells indicate unknown mutation status (not analyzable).

Patient	APC	CTNNB1	KRAS	TP53
1	wt	mut	wt	wt
2				
3	mut	wt	wt	wt
4	mut	wt	mut*	wt
5	mut	wt	wt	wt
6				
7	wt	wt	wt	wt
8	mut	wt	wt	wt
9	wt	wt	wt	wt
10	wt	wt	wt	wt
11	mut	wt	wt	wt
12	mut	wt	wt	wt
13				
14	mut	wt	mut*	wt
15	wt	wt	wt	wt
16				
17	wt	wt	wt	wt
18		mut	wt	
19		wt	wt	
20		mut	wt	
21.a		mut	wt	
21.b	mut	wt	wt	wt
22		wt	wt	
23				
24		wt	mut	
25	wt	mut	wt	wt
26	mut	wt	wt	wt
27	mut	wt	wt	wt

*** *KRAS* mutations not affecting codon 12/13.**
